# The Rise of mRNA COVID-19 Vaccine-Associated Myocarditis and Its Implications on the Future Use of This New Vaccine Platform

**DOI:** 10.7759/cureus.25631

**Published:** 2022-06-03

**Authors:** Jamal A Anthony, Tatiana Echeverry, Robert D Fishberg

**Affiliations:** 1 Internal Medicine, Overlook Medical Center-Atlantic Health System, Summit, USA; 2 Cardiology, Overlook Medical Center-Atlantic Health System, Summit, USA

**Keywords:** vaccine-associated myocarditis, vaccine, mrna, myocarditis, covid-19

## Abstract

Vaccine-associated myocarditis is becoming increasingly documented as a complication of the messenger ribonucleic acid (mRNA) vaccination platform. This complication so far has been found to predominantly affect the younger male population within seven days of receiving the second dose of an mRNA vaccine. We present a case of a 45-year-old male found to have clinical, biochemical, and radiological evidence of myocarditis three days after receipt of the second dose of the Moderna COVID-19 vaccine. Troponin I and inflammatory marker trends, in addition to the use of cardiac MRI imaging, was important in making the diagnosis. Symptom resolution was achieved after two months of colchicine and anti-heart failure medications. We highlight the occurrence of this rare vaccine complication in an endeavor to stress the need for further research to better understand this condition so that better guidance can be provided to the medical community on how best to screen and manage it.

## Introduction

Myocarditis is the inflammation of the heart muscle and pericarditis is inflammation of the surrounding lining of the heart. Myocarditis is an uncommon condition with an estimated incidence of 10-20 cases per 100,000 persons [1].The causes can be infectious and non-infectious. Viral myocarditis is the most common cause in the United States (US) and other developed countries [1,2].

The clinical presentation of myocarditis can be variable. Patients can present with mild symptoms, such as generalized fatigue, malaise, and chest pain, whereas in severe cases known as fulminant myocarditis, this inflammation can be significant and lead to a decrease in the heart’s ability to effectively pump blood to the rest of the body. Hence, patients can present with congestive heart failure, arrhythmias, cardiogenic shock, and even cardiac arrest in extreme cases [1-3].

Cardiac enzymes are typically elevated. However, negative troponin I does not rule out the condition [2]. Electrocardiogram findings tend to be variable, with some cases showing non-specific changes, to others indicating overt ST-segment elevations. Though diagnosis can be made on clinical presentation it is classically confirmed by an endomyocardial biopsy. However, more recently with the advent of the cardiac MRI, specific criteria known as Lake Louise criteria can be applied to confirm the diagnosis without the need for biopsy. Usually, myocarditis on cardiac MRI is revealed by late gadolinium enhancement and elevated native T1 and T2 [2,4,5].

In the past, myocarditis was a more rarely documented complication of vaccination. However, with the advent of the new messenger ribonucleic acid (mRNA) vaccines, vaccine-associated myocarditis (VAM) is starting to take some of the shine as prominent cause of myocarditis. In the US VAM has an incidence of ~4.8 cases per million after second dose of mRNA vaccines [1,2,5]. This is especially prominent in the younger male population, which warrants further discussion and investigation. In this report we highlight the unexpected occurrence of myocarditis in a young male three days after receiving the second dose of the mRNA-1273 (Moderna) vaccine and discuss why such findings are important for the future of mRNA vaccines.

## Case presentation

A 45-year-old male with no significant past medical history presented for acute-onset sharp mid-sternal chest pain that woke him from sleep at 2:30 am on the day of presentation. The pain was associated with dyspnea and diaphoresis. Of note, he received the second dose of the Moderna COVID-19 vaccine three days prior to presentation. Physical examination was unremarkable, and vitals were stable.

**Figure 1:**
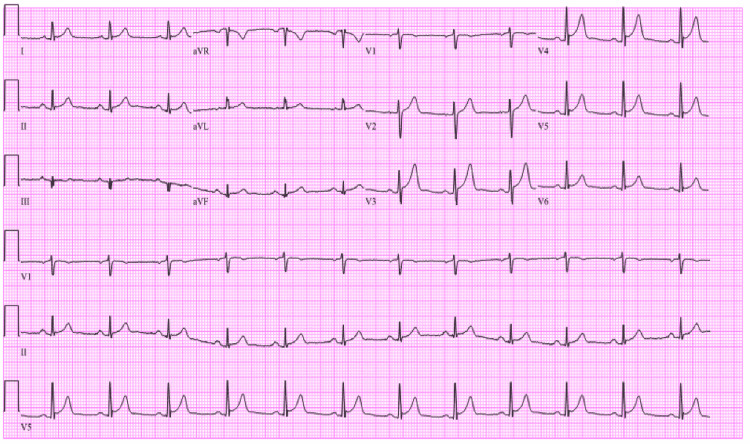
Initial EKG showing ST-segment elevations in V2-V4

COVID-19 polymerase chain reaction (PCR) was negative, and the complete blood count and complete metabolic panel were unremarkable. Initial EKG showed ST elevations in leads V2-V4 (Figure 1) and troponin I was 4.37 (Table 1). Emergent cardiac catheterization was done and showed minimal non-obstructive disease and mildly reduced systolic function with an ejection fraction (EF) of 50%. 

**Table 1:** Trend of troponin I and CRP throughout disease course CRP, C-reactive protein

Disease timeline	Admission	Admission lab results	8 hours after admission	12 hours after admission	20 hours after admission	Persistence of chest pain prompting repeat echocardiogram and trending of CRP	24 hours after admission	48 hours after admission	Discharge	2 weeks after discharge	4 months after discharge	5 months after discharge
Troponin I ng/mL		4.370	5.470	6.800	7.360		5.930	4.20		4	3	-
CRP mg/dL		-	-	-	-		25.8	13.3		2.3	2.7	2.2

**Figure 2:**
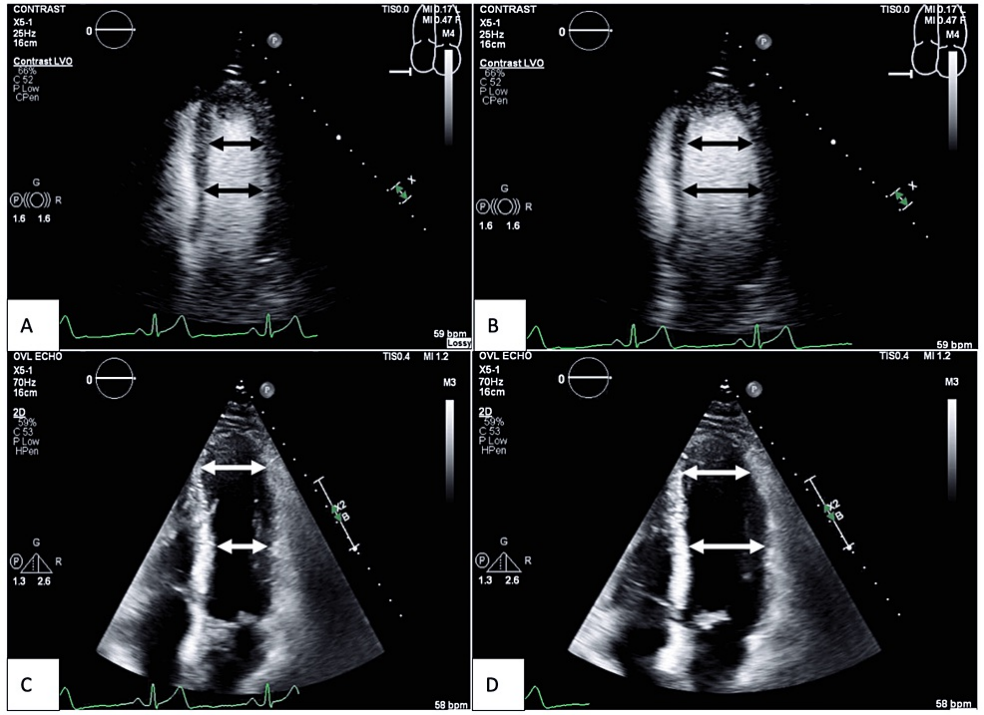
Apical four-chamber view of first follow-up echocardiogram after cardiac catheterization A: Contrast study showing the left ventricle in systole; B: contrast study showing left ventricle in diastole; C: non-contrast study showing the left ventricle in systole; D: non-contrast study showing the left ventricle in diastole. Note that both contrast and non-contrast studies revealed good contraction of the mid-segment of the left ventricle but the diameter (the length of white and black double-headed arrows) of the apical segment in diastole and systole does not significantly change. This demonstrated apical hypokinesis. Ejection fraction had decreased to 40%.

He was determined to have possible stress-induced cardiomyopathy and treatment with carvedilol was started. However, his symptoms continued to persist. A follow-up echocardiogram showed an EF of 40% with mild hypokinesis in the apical segments (Figure 2). Inflammatory markers were ordered and revealed increasing C-reactive protein (Table 1). He was started on colchicine. Symptoms resolved and he was discharged with instructions to avoid strenuous activity.

**Figure 3:**
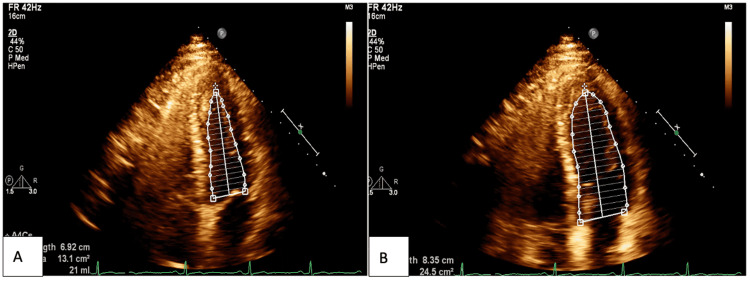
Apical four-chamber view of second follow-up echocardiogram after cardiac catheterization done after two weeks of treatment with colchicine A: Non-contrast study showing the left ventricle in systole; B: non-contrast study showing left ventricle in diastole. Note how there is now good contraction of all segments of the left ventricle when compared to Figure 2, indicating resolution of the apical hypokinesis. Ejection fraction improved to 60%.

**Figure 4:**
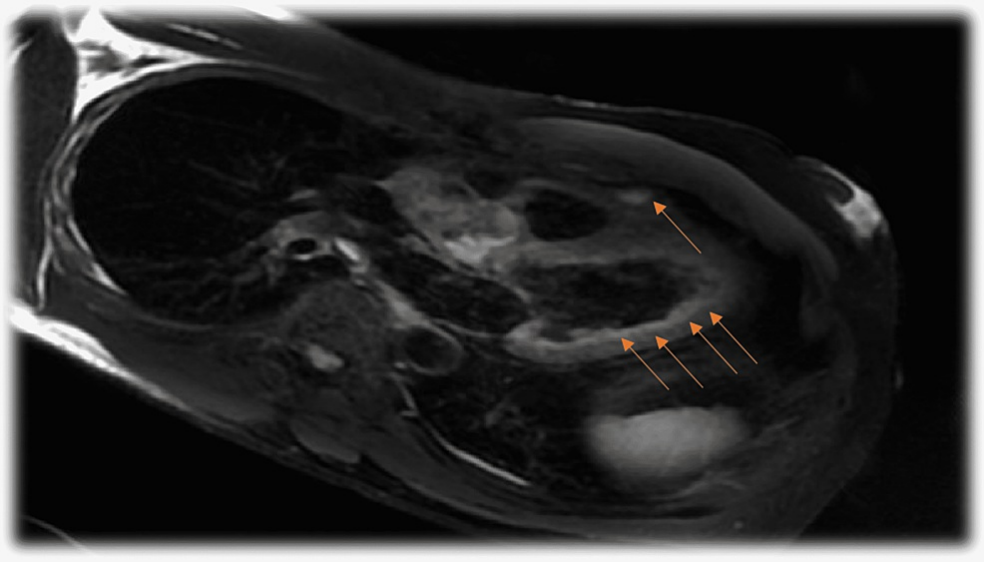
Cardiac MRI showing multifocal myocardial late gadolinium enhancement (arrows) consistent with myocarditis

The patient had a follow-up appointment two weeks after discharge and had a slight improvement in symptoms. An angiotensin-converting enzyme inhibitor (ACEI) was added to the regimen. An echocardiogram was repeated and it showed an improved EF of 60% and resolved apical hypokinesis (Figure 3). The patient had a second follow-up two weeks later and had persistent lightheadedness. Holter monitoring was done without arrhythmias. Cardiac MRI showed multifocal mid-myocardial and subepicardial late gadolinium enhancement in apical and basal segments. T2 mapping did not show myocardial edema. These findings were consistent with resolving/prior myocarditis (Figure 4). He was continued on colchicine, ACEI, and beta-blocker. After two months, the patient was tolerating exercise without symptoms. However, he had further follow-up visits four and five months after discharge for mild intermittent chest discomfort and dizziness. Troponin I and CRP remained normal. The Follow-up stress test was negative. ACEI was held and he continued beta-blocker and colchicine. He has since improved. Colchicine, beta-blockers, and ACEI were discontinued six months after discharge. 

## Discussion

Three vaccines have been given emergency use authorization in the US and they employ different biochemical methods to achieve the similar goal of stimulating the immune system to produce neutralizing antibodies that provide protection against the COVID-19 virus [6]. Two of the vaccines used in the US employ the mRNA vaccine platform: BNT162b2 (Pfizer-BioNTech) and the mRNA-1273 (Moderna) vaccines, while the Ad26.COV2.S (Jenssen) vaccine uses the COVID-19 adenovirus vector platform [6]. According to the Center for Disease Control (CDC) [3], while there have been increasing reports of VAM with the use of mRNA vaccines, there is no evidence to suggest similar complications with the Jenssen vaccine (though this has its separate complications as well). Our patient was a young adult male with no prior history of heart disease, viral infection, or COVID-19 who developed signs and symptoms consistent with myocarditis three days after his second dose of the mRNA-1273 vaccine.

Bozkurt et al. [5] highlighted data obtained from the CDC and Prevention Advisory Committee on immunization practices through the Vaccine Adverse Events Reporting System, which demonstrated an exponential increase in the number of expected vs. observed cases of post-vaccination myocarditis in males across an age range starting from 12 years, where the observed numbers were estimated to be >30 times higher than expected, to 65+ where the numbers were estimated 3 times less than expected. The data showed a gradual decrease in the difference between expected and observed as age increased. Further stratification of this data by Bozkurt et al. revealed that differences between expected and observed reports of myocarditis in females were remarkably lower when compared to affected males of the same age groups. Based on this data our patient being a 45-year-old male was 2-17 times more likely to get myocarditis after vaccination with an mRNA vaccine. Had he been 12-17 years old this would have made his chances even higher.

Recently published data from the active national surveillance from the Israeli Ministry of Health National Database demonstrated a higher incidence of myocarditis among the Israeli population who received two doses of the BNT162b2 mRNA vaccine [6,7]. Mevorach et al. [7] reported that from the 5 million vaccinated Israeli residents, a rate of post-vaccination myocarditis of approximately 1 per 26,000 males and 1 per 218,000 females after the second vaccine dose was identified with the highest risk again among young male recipients similar to what is being reported in the US by the CDC for both mRNA vaccines [1,2].

Some may argue that the data involving an entire nation may be confounded by patients with comorbidities that may be contributing to more exaggerated side effects after vaccination. However, a recent retrospective case series study on a group of perfectly healthy US military members would not support such an argument. According to Montgomery et al. [8], all military members were previously healthy with a high level of fitness. Approximately 70% received the mRNA-1273 vaccine (like our patient) and ~87% had symptom onset following the second dose of an appropriately spaced two-dose series. Like our patient, these patients in the study had significantly elevated cardiac troponin levels, and all who underwent cardiac MRI within the acute phase of illness had findings consistent with the clinical diagnosis of myocarditis. This study concluded that the number was higher than expected among the male military members after a second vaccine dose [8]. Such findings are consistent with the data reported in the Israeli study and that reported by the CDC.

Although VAM has been well documented historically with the use of the older vaccines, such as smallpox and hepatitis B, the number of vaccine-related cases reported has never been this high, even in the younger male population [5,9]. Due to the lack of myocardial biopsy performance for this often-self-limiting condition, the exact pathogenesis of VAM is still poorly understood. However, several mechanisms have been proposed.

One proposed mechanism is through molecular mimicry. Antibodies against the spike proteins produced from the translation of the mRNA in host cells have been shown to cross-react with human polypeptides that are structurally similar [10]. One such peptide protein sequence is alpha-myosin found in the heart. However, one may argue that if this were the case the incidence of VAM would be similar in patients who received the adenovirus vector-based vaccines. Perhaps it may be more prominent in mRNA vaccines as the mRNA itself is highly immunogenic and may in addition cause direct cell damage to the myocardium. When the mRNA particles are exposed to antigen-presenting dendritic cells, for example, this may lead to the production of cytokines in certain individuals who are more sensitive to the presence of the modified mRNA [5,10].

The reasons may be due to a particular genetic predisposition not yet identified which may cause some individuals to be affected while others are not. Most recently the lipid nanoparticles used to carry the mRNA to the target host cells have been considered as well. Though this technology has been present for several years being employed in chemotherapeutic agents, some argue that the combination of the lipid nanoparticles used with the mRNA may be inducing a hypersensitivity reaction and eosinophilic myocarditis [5,10,11]. Kounis et al. [11] reported two cases of biopsy-confirmed eosinophilic myocarditis two weeks after receipt of the BNT162b2 mRNA vaccine which supports this theory. However, more frequent biopsies in cases of mRNA VAM would need to be done and reported to conduct more research to validate this proposed mechanism.

Finally, the answer to the question of why there is a younger male predominance for VAM is also unclear. However, it is well known that estrogen has anti-inflammatory properties as it inhibits pro-inflammatory T-cells, resulting in a dampening of the cell-mediated immune response [5,10]. Testosterone, on the other hand, is known to have pro-inflammatory effects as it inhibits anti-inflammatory cells and promotes the commitment of T-helper 1- type immune responses [5,10]. Hence, it is reasonably postulated that the variation in the levels of particular sex hormones with age and sex may explain the increased incidence of VAM in younger males. However, definitive answers to these burning questions and evidence for these theories can only be provided with the continued research on this matter. Only then can more light be shone on the enigmas of mRNA VAM.

## Conclusions

mRNA vaccines have saved billions of lives since being introduced in December 2020. VAM is self-limiting in most cases. Therefore, we believe that the benefits of mRNA vaccines outweigh the risks of COVID-19 infection. However, there is growing evidence to suggest a causal relationship between mRNA vaccines and myocarditis as demonstrated in this case which cannot be ignored. Given the mRNA vaccine platform is set to possibly monopolize the future of vaccine production in years to come, we believe that the prevalence of cases will continue to increase. Hence, resources need to be allocated to funding studies geared toward understanding the pathophysiological mechanisms behind mRNA VAM. Furthermore, the development of guidelines for screening at-risk demographic groups and appropriate management protocols to avoid fatalities and/or complications from unidentified/untreated cases are also needed; this way the mRNA vaccine platform can be safely implemented for future vaccines.
